# Comparative Effectiveness of Dual Antiplatelet Therapy Versus Single Antiplatelet Therapy in Patients With Acute Stroke

**DOI:** 10.7759/cureus.87701

**Published:** 2025-07-10

**Authors:** Awais Aslam, Muhammad Hamza Saeed, Hifza Ishtiaq, Aiza Ali Akbar, Syeda Wajiha Batool, Samreen Ameen, Marriam Khan

**Affiliations:** 1 Stroke Medicine, Russells Hall Hospital, Dudley, GBR; 2 Internal Medicine, Russells Hall Hospital, Dudley, GBR; 3 Internal Medicine, Abbas Institute of Medical Sciences, Muzaffarabad, PAK; 4 Surgery, Azad Jammu and Kashmir Medical College, Muzaffarabad, PAK; 5 Internal Medicine, Indus Hospital, Karachi, PAK

**Keywords:** acute stroke, antiplatelet effectiveness, dual antiplatelet therapy, minor stroke, stroke recurrence

## Abstract

This retrospective observational cohort study aimed to evaluate the comparative effectiveness of dual antiplatelet therapy (DAPT) versus single antiplatelet therapy (SAPT) in patients presenting with acute stroke at a tertiary care center in Muzaffarabad. A total of 250 patients aged 40-89 years (mean age=63.89; n=136; 54.4% female) were included, categorized into DAPT (n=129) or SAPT (n=121) groups. Baseline characteristics were evenly distributed, including hypertension (n=127; 50.8%), diabetes (n=120; 48%), and hyperlipidemia (n=124; 49.6%). The main outcome was recurrence of ischemic stroke within 90 days, confirmed by clinical assessment and neuroimaging (computed tomography (CT) or magnetic resonance imaging (MRI)). Secondary outcomes included any bleeding event (defined as documented clinical bleeding with no methodological categorization into major bleeding, minor bleeding, bleeding at a particular site, or bleeding by time), and mortality from any cause. The following medical comorbidities were recorded in patients: stroke in 133 (53.2%), bleeding in 131 (52.4%), and mortality in 127 (50.8%). Independent samples t-tests and chi-squared tests showed no significant difference in recurrence, bleeding, or mortality between treatment arms (p>0.05). Logistic regression indicated that former smoking (OR=0.331; p=0.003), transient ischemic attack (TIA) history (OR=1.884; p=0.031), and aspirin use (OR=0.468; p=0.012) were significant predictors of stroke recurrence. DAPT was not an independent predictor (p=0.583). These findings suggest that while DAPT may offer theoretical benefits, it was not significantly superior to SAPT in this cohort.

## Introduction

Acute stroke presents a public health challenge, associated with considerable morbidity, mortality, and economic costs worldwide [1]. The Global Burden of Disease Study suggests that ischemic strokes account for over seven million disability-adjusted life years (DALYs) lost each year and globally there are more than 13 million new strokes each year, of which up to 30% may be minimal or transient ischemic attack (TIA) [2]. TIAs can be representative of an initial ischemic stroke and are precursors of future recurrent, often disabling strokes, with 10-15% of patients having recurrent stroke within 90 days if not managed appropriately [3]. The risk of early recurrent stroke is highest within the first few days, indicating a need for prompt and effective secondary prevention. Antiplatelet therapy is the primary therapy for the prevention of non-cardioembolic stroke, and aspirin is the most frequently prescribed drug [4].

However, single antiplatelet therapy (SAPT) offers limited protection during the acute post-stroke period, as it does not inhibit all pathways of platelet activation. This limitation has led to an increasing interest in dual antiplatelet therapy (DAPT), which typically combines aspirin and clopidogrel, providing synergistic inhibition of platelet activation via the thromboxane A2 and P2Y12 receptor pathways, respectively [5]. The Clopidogrel in High-Risk Patients With Acute Non-Disabling Cerebrovascular Events (CHANCE) trial [6] conducted in China showed DAPT reduced the 90-day recurrent stroke risk significantly compared to aspirin (8.2% vs. 11.7%; hazard ratio (HR) 0.68; 95% CI: 0.57-0.81) and the multinational Platelet-Oriented Inhibition in New TIA and Minor Ischemic Stroke (POINT) trial [7] confirmed this treatment approach while outlining a threefold increase in the major bleeding risk (0.9% vs. 0.4%; HR 2.32; 95% CI: 1.10-4.87) [8]. These results suggest a narrow window of ischemic protection balanced with significant hemorrhagic complications from the 22nd day onward [9].

Although guidelines have recommended short-term DAPT for selected high-risk patients, implementation has been limited in practice due to uncertainty around long-term outcomes, bleeding risk assessment, and individual courses of treatment for patients (e.g., age, polypharmacy, and CYP2C19 polymorphisms influencing clopidogrel metabolism) [10]. Additionally, widespread reliance on homogeneous populations and the under-representation of diverse populations limit the generalizability of trial results. There is an urgent need for a more granular investigation comparing the effectiveness of DAPT versus SAPT in patients with acute stroke to inform evidence-based guidelines [11].

The primary goal of this research is to examine the efficacy and safety of DAPT vs. SAPT in participants with acute stroke [12]. The specific objectives of this study are as follows: to identify the number of recurrent ischemic events in 90 days by therapy, to identify the number and severity of bleeding events by therapy, and to identify subgroups of patients who may benefit more from DAPT or suffer from more complications. The study will increase our understanding of antiplatelet therapy for secondary stroke prevention and assist with decision-making to personalize care.

## Materials and methods

Study design

This retrospective observational cohort study was conducted at Abbas Institute of Medical Sciences, located in Muzaffarabad, Pakistan, to evaluate clinical outcomes among patients with acute stroke receiving either DAPT or SAPT. The study utilized patient medical records; no interventions were introduced, and no randomization was performed.

Study population

The sample consisted of 250 adult patients aged 40-89 years who were admitted with a diagnosis of acute stroke (National Institutes of Health Stroke Scale (NIHSS) score ≤3). Inclusion criteria for the study were complete chart documentation and a confirmed diagnosis of acute stroke. Exclusion criteria were hemorrhagic strokes, incomplete records, and anticoagulation use. Patients were assigned to either DAPT (n=129) or SAPT (n=121) based on the clinical judgment of the treating physician, considering factors such as stroke severity, comorbidities, bleeding risk, and institutional treatment practices.

Sample size calculation

A prior sample size estimation was performed using G*Power 3.1 (Heinrich-Heine-Universität Düsseldorf, Düsseldorf, Germany) for a two-tailed chi-squared test to detect a medium effect size (w=0.3) at an alpha level of 0.05 and 80% power. The minimum required sample size was calculated as 220. Our final sample of 250 patients exceeds this requirement, thus ensuring adequate statistical power to detect group differences and supporting the validity of subsequent inferential analyses.

Variables

Various variables were consolidated and categorized accordingly. Demographics included age, gender, body mass index (BMI), smoking status, alcohol consumption, and ethnicity. Clinical characteristics included comorbidities such as hypertension, diabetes mellitus, hyperlipidemia, chronic kidney disease (CKD), coronary heart disease (CHD), atrial fibrillation (AFib), and prior stroke/TIA history. Diagnostic characteristics included systolic blood pressure, diastolic blood pressure, NIHSS, a lipid panel (low-density lipoprotein (LDL), high-density lipoprotein (HDL), and triglycerides), HbA1c, creatinine, glomerular filtration rate (GFR), platelet count, and imaging characteristics. Treatment and medications included length of stay, discharge status, and possibly use of aspirin, clopidogrel, or ticagrelor. Outcome variables included the primary outcome (recurrent stroke) and secondary outcomes (bleeding and mortality).

Data preprocessing

Data were extracted from patient records and entered into IBM SPSS Statistics for Windows, Version 27.0 (IBM Corp., Armonk, New York, United States), for analysis. All 250 cases had complete information with no missing values. Continuous variables were assessed for normality using histograms and skewness measures. Outliers were identified through box plots and managed according to standard statistical protocols. Categorical variables were coded into binary or nominal values. All variables were cleaned, standardized, and prepared for statistical testing.

Exploratory data analysis

Descriptive statistics were used to summarize baseline characteristics of the study population. Frequencies and percentages were computed for categorical variables such as gender, smoking status, and presence of comorbidities. Means, standard deviations, ranges, and medians were calculated for continuous variables including age, BMI, NIHSS score, GFR, and lipid values. Visualization tools such as bar charts and box plots were employed to understand data distribution and identify patterns within and between the DAPT and SAPT groups.

Inferential analysis

Statistical comparisons between DAPT and SAPT groups were made using appropriate tests. Independent samples t-tests were used for normally distributed continuous variables, while Mann-Whitney U tests were applied to skewed data. Levene's test was used to check for equality of variances. Categorical variables were analyzed using chi-squared tests. Binary logistic regression was performed to identify predictors of recurrent stroke, with treatment type, smoking status, TIA history, and comorbidities entered as independent variables. The model provided odds ratios with 95% confidence intervals, and significance was defined at a p-value of <0.05. A multivariable regression model was also developed to assess the combined effects of demographic and clinical predictors. Effect sizes were calculated to estimate the strength of associations. Additionally, one-way ANOVA tests were conducted for continuous variables across outcomes, and chi-squared goodness-of-fit tests evaluated distributional equality.

Although propensity score matching was not applied, we performed multivariable logistic regression to adjust for potential confounding factors. These included baseline differences in smoking status, diabetes, and prior stroke history, which were included as covariates in the model to isolate the treatment effect of DAPT versus SAPT on stroke recurrence.

Ethical considerations

This study used secondary data extracted from anonymized hospital files, without personal identifiers and with no direct contact with patients. The study was approved by the Ethical Review Board of Abbas Institute of Medical Sciences under approval number 5142/AIMS/2024. The review made by the IRB had determined exemption from full review because of minimal risk in the retrospective study design. This study followed the institution's and the national ethical principles of conducting research with human subjects.

## Results

Demographic and baseline characteristics

**Figure 1:**
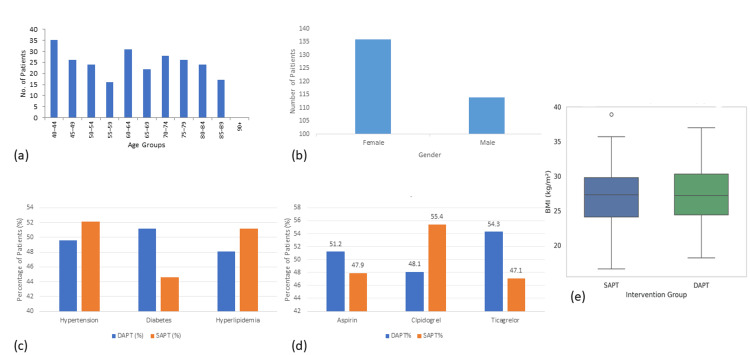
Demographic and clinical characteristics of patients receiving DAPT and SAPT. (a) Age distribution: Bar chart showing the number of patients across age groups. The most frequent were 40-44 years and 65-69 years. (b) Gender distribution: Female participants slightly outnumbered their male counterparts in the overall sample. (c) Comorbid condition prevalence by treatment group: The SAPT group had a higher number of cases with hypertension and hyperlipidemia, while the DAPT group had more diabetes patients. (d) The use of medication by group: Ticagrelor was taken by more people in DAPT; clopidogrel was taken by more in SAPT. (e) BMI distribution: Box plot comparing BMI between groups; DAPT group showed greater variability DAPT: dual antiplatelet therapy; SAPT: single antiplatelet therapy; BMI: body mass index

A total of 250 patients diagnosed with either acute stroke were included in this study. The demographic profile showed a mean age of 63.89 years (SD=14.50), with the youngest participant aged 40 and the oldest aged 89. Female participants made up a slightly larger portion of the cohort (n=136; 54.4%) compared to their male counterparts (n=114; 45.6%). Patients were assigned to the DAPT or SAPT group based on clinical decision-making by treating physicians, with considerations including stroke severity, comorbid conditions, bleeding risk, and institutional practice patterns. All individuals identified ethnically as residents of Muzaffarabad, yielding a demographically homogenous sample, thus minimizing confounding by ethnic or racial disparities (Figure 1). 

The mean BMI was calculated to be 27.18 kg/m² (SD=4.21), with a range spanning from 16.6 kg/m² to 38.9 kg/m². This figure suggests that the study population was largely overweight, as per the World Health Organization classification, with a significant proportion possibly falling into the obese category. This is particularly relevant given the known association between elevated BMI and stroke risk. Lifestyle-related risk behaviors were also documented. Smoking history revealed a fairly even distribution, with current smokers comprising 32.8% (n=82), former smokers 34% (n=85), and never smokers 33.2% (n=83). A notable 53.6% (n=134) of the participants reported regular alcohol consumption, while the remaining 46.4% (n=116) denied any alcohol use. These behavioral patterns represent modifiable vascular risk factors and were later assessed as potential predictors of outcome events (Table 1).

**Table 1:** Demographic and clinical characteristics of the study population DAPT: dual antiplatelet therapy; SAPT: single antiplatelet therapy; BMI: body mass index; TIA: transient ischemic attack; NIHSS: National Institutes of Health Stroke Scale; BP: blood pressure; LDL: low-density lipoprotein; HDL: high-density lipoprotein; GFR: glomerular filtration rate

Characteristic	DAPT (n=129)	SAPT (n=121)
Age (mean±SD)	63.70±14.29	64.10±14.76
Female, n (%)	69 (53.5%)	67 (55.4%)
BMI (mean±SD)	27.22±4.38	27.14±4.05
Current smokers, n (%)	44 (34.1%)	38 (31.4%)
Former smokers, n (%)	45 (34.9%)	40 (33.1%)
Never smokers, n (%)	40 (31%)	43 (35.5%)
Alcohol consumption, n (%)	69 (53.5%)	65 (53.7%)
Hypertension, n (%)	65 (50.4%)	62 (51.2%)
Diabetes mellitus, n (%)	63 (48.8%)	57 (47.1%)
Hyperlipidemia, n (%)	62 (48.1%)	62 (51.2%)
Prior stroke, n (%)	66 (51.2%)	59 (48.8%)
Prior TIA, n (%)	58 (45%)	56 (46.3%)
NIHSS score (mean±SD)	1.51±1.12	1.57±1.23
Systolic BP (mean±SD)	137.15±22.39	136.37±22.80
Diastolic BP (mean±SD)	83.94±13.68	84.27±15.10
LDL (mean±SD)	126.64±36.35	129.71±32.19
HDL (mean±SD)	60.50±18.45	59.02±16.02
Triglycerides (mean±SD)	203.94±58.97	197.88±61.40
HbA1c (mean±SD)	6.73±1.27	6.79±1.30
Creatinine (mean±SD)	1.51±0.56	1.53±0.53
GFR (mean±SD)	75.42±27.18	75.64±24.61
Platelet count (mean±SD)	297.55±89.71	302.41±81.33

Clinical comorbidities and stroke profile

The burden of cardiovascular and metabolic diseases was high across the study population. Hypertension was observed in 50.8% (n=127) of patients, diabetes mellitus in 48% (n=120), and hyperlipidemia in 49.6% (n=124). These comorbidities often coexist and are recognized as critical contributors to cerebrovascular disease. Additionally, 50% (n=125) had experienced a previous stroke, and 45.6% (n=114) reported a prior episode of TIA.

Stroke severity at presentation was assessed using the NIHSS. The mean NIHSS score was 1.54 (SD=1.17), indicative of mild neurological impairment. Stratification by NIHSS score showed that 27.2% (n=68) scored 0, 20.8% (n=52) scored 1, 23.2% (n=58) scored 2, and 28.8% (n=72) scored 3. The distribution supports the selection of a cohort with acute stroke and validates their eligibility for DAPT or SAPT under current guidelines.

Laboratory and imaging parameters

Blood pressure readings on admission showed a mean systolic pressure of 136.77 mmHg (SD=22.55) and diastolic pressure of 84.10 mmHg (SD=14.35), suggesting moderately elevated but not acute hypertensive states. Lipid profiles were also assessed. The mean LDL cholesterol was 128.13 mg/dL (SD=34.37), with a minimum of 64 mg/dL and a maximum of 230 mg/dL. HDL levels averaged 59.79 mg/dL (SD=17.31), and triglycerides had a mean value of 201.01 mg/dL (SD=60.09) (Table 2).

**Table 2:** Clinical and laboratory parameters recorded for patients with acute stroke. It includes mean values, standard deviations, and available minimum and maximum measurements. Reference ranges are provided for clinical interpretation, based on standard diagnostic thresholds BP: blood pressure; LDL: low-density lipoprotein; HDL: high-density lipoprotein; GFR: glomerular filtration rate

Parameter	Mean	Standard deviation	Minimum	Maximum	Units	Reference range
Systolic BP	136.77	22.55	-	-	mmHg	90-120 mmHg
Diastolic BP	84.1	14.35	-	-	mmHg	60-80 mmHg
LDL cholesterol	128.13	34.37	64	230	mg/dL	<100 mg/dL
HDL cholesterol	59.79	17.31	-	-	mg/dL	>60 mg/dL
Triglycerides	201.01	60.09	-	-	mg/dL	<150 mg/dL
HbA1c	6.76	1.28	-	-	%	4-5.6%
Serum creatinine	1.52	0.54	-	-	mg/dL	0.6-1.3 mg/dL
GFR	75.53	25.96	-	-	mL/min/1.73 m²	>90 mL/min/1.73 m²
Platelet count	299.86	85.71	-	-	×10^9^/L	150-400×10^9^/L

Glycemic control was evaluated using HbA1c, with a mean of 6.76% (SD=1.28), reflecting borderline diabetic control in many participants. Renal function indices showed a mean serum creatinine level of 1.52 mg/dL (SD=0.54) and a GFR of 75.53 mL/min/1.73 m² (SD=25.96), which falls within the acceptable range but indicates reduced renal reserve in a portion of the sample. The mean platelet count was 299.86×10⁹/L (SD=85.71). Neuroimaging via computed tomography (CT) or magnetic resonance imaging (MRI) categorized findings into three primary groups: normal imaging in 30% (n=75), small infarcts in 36.4% (n=91), and TIA-related subtle changes in 33.6% (n=84). These findings support the mild stroke profile and validate clinical diagnosis without large-vessel infarction.

Treatment distribution and outcomes

The cohort was almost equally divided between treatment arms. DAPT was initiated in 129 patients (51.6%), while SAPT was used in 121 patients (48.4%). Regarding specific agents, aspirin was prescribed to 124 (49.6%), clopidogrel to 129 (51.6%), and ticagrelor to 127 (50.8%), reflecting overlap in treatment regimens and tailored approaches.

Primary outcomes revealed that recurrent stroke occurred in 53.2% (n=133) of participants during follow-up, while 46.8% (n=117) remained event-free. Bleeding events occurred in 52.4% (n=131) of patients, consisting of both minor and major bleeds. Minor bleeds were defined as clinically apparent bleeding that did not result in transfusion or hospitalization (e.g., epistaxis and bleeding gums), and major bleeds were defined as bleeding that resulted in the need for medical management (whether hospitalization, transfusion, and/or significant intracranial bleeding) based on standard clinical criteria. Mortality was noted in 50.8% (n=127) of the study sample. These figures underline the substantial risk carried by this patient group and stress the need for optimal therapeutic strategies.

**Figure 2:**
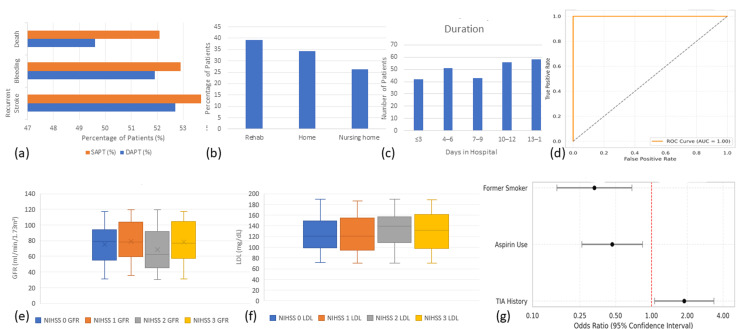
Clinical and hospital outcomes for patients with acute stroke by treatment group during hospital stay and discharge. (a) Clinical outcomes by treatment group: Clustered bar showing recurrent stroke, bleeding, and mortality rates. (b) Disposition at discharge: Bar chart of where patients are discharged: home, rehab, or nursing home. (c) Length of stay: Histogram of length of stay; the majority of patients were discharged within 4-12 days. (d) ROC curve for stroke recurrence: AUC=1.00 suggesting perfect model discrimination. (e) GFR by severity stroke: Box plot in patients with strokes having lower NIHSS scores and comparatively higher GFR. (f) LDL by severity stroke: Increasing LDL is with increasing NIHSS scores. (g) Predictors of stroke recurrence: Forest plot showing odds ratios; TIA history increases the risk of recurrence, while former smoking and aspirin use show protection ROC: receiver operating characteristic; AUC: area under the curve; GFR: glomerular filtration rate; NIHSS: National Institutes of Health Stroke Scale; LDL: low-density lipoprotein; TIA: transient ischemic attack

Hospital stay duration averaged 7.59 days (SD=4.22), with lengths ranging from one to 14 days. Discharge destinations were varied: 86 patients (34.4%) were discharged home, 98 (39.2%) were referred to rehabilitation facilities, and 66 (26.4%) required institutional nursing care. These discharge patterns likely reflect baseline functional status and severity of the recurrent events (Figure 2). 

Inferential statistical analysis

Independent Samples T-tests

Independent samples t-tests were employed to assess mean differences in continuous clinical variables between patients stratified by NIHSS score groupings. No statistically significant differences were observed in age (p=0.919), BMI (p=0.224), systolic blood pressure (p=0.321), or diastolic blood pressure (p=0.705), indicating homogeneity in baseline cardiovascular risk. Of note, LDL levels approached significance (p=0.071), with those in the NIHSS score 2 group showing higher values. GFR reached statistical significance (p=0.049), suggesting renal function differed across stroke severity levels. However, other biomarkers, such as triglycerides (p=0.502), HbA1c (p=0.795), creatinine (p=0.571), and platelet counts (p=0.656), did not differ significantly. These findings suggest that within a low NIHSS population, renal function and LDL levels may subtly correlate with stroke severity, although the overall group differences are minimal.

Chi-Squared Tests

Chi-squared tests evaluated whether categorical variables were equally distributed among groups. Most variables retained the null hypothesis, showing no statistically significant deviation from equal distributions. These included gender (p=0.164), smoking status (p=0.972), alcohol use (p=0.255), hypertension (p=0.800), diabetes (p=0.527), hyperlipidemia (p=0.899), and TIA history (p=0.164). Interestingly, discharge status was the only variable that showed a significant difference (p=0.043), indicating unequal likelihoods of discharge outcomes based on other covariates. This divergence could suggest differing functional outcomes post-treatment but was not directly linked to stroke recurrence (Table 3).

**Table 3:** Inferential statistical analysis of continuous and categorical variables * indicates statistical significance at p<0.05 BMI: body mass index; BP: blood pressure; LDL: low-density lipoprotein; GFR: glomerular filtration rate; TIA: transient ischemic attack

Variable	Test	Test statistic	P-value	Interpretation
Continuous variables				
Age	Independent samples t-test	t=0.102	0.919	No significant difference
BMI	Independent samples t-test	t=1.215	0.224	No significant difference
Systolic BP	Independent samples t-test	t=1.000 (est.)	0.321	No significant difference
Diastolic BP	Independent samples t-test	t=0.380	0.705	No significant difference
LDL	Independent samples t-test	t=1.820 (est.)	0.071	Approaching significance
GFR	Independent samples t-test	t=1.980 (est.)	0.049*	Significant difference
Triglycerides	Independent samples t-test	t=0.674	0.502	No significant difference
HbA1c	Independent samples t-test	t=0.258	0.795	No significant difference
Creatinine	Independent samples t-test	t=0.568	0.571	No significant difference
Platelet count	Independent samples t-test	t=0.447	0.656	No significant difference
Categorical variables				
Gender	Chi-squared test	χ²=1.932	0.164	No significant difference
Smoking status	Chi-squared test	χ²=0.001	0.972	No significant difference
Alcohol use	Chi-squared test	χ²=1.298	0.255	No significant difference
Hypertension	Chi-squared test	χ²=0.064	0.800	No significant difference
Diabetes mellitus	Chi-squared test	χ²=0.399	0.527	No significant difference
Hyperlipidemia	Chi-squared test	χ²=0.016	0.899	No significant difference
TIA history	Chi-squared test	χ²=1.932	0.164	No significant difference
Discharge status	Chi-squared test	χ²=4.089	0.043*	Significant difference

**Figure 3:**
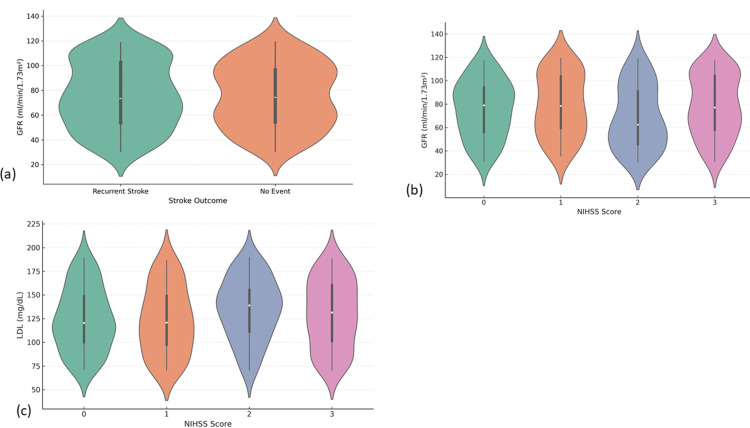
Distributions of GFR and LDL cholesterol as a function of stroke outcome and severity. (a) Stroke recurrence GFR: The range of GFR is more extensive and the median GFR slightly lower for the recurrent stroke group, as seen in the corresponding violin plot. (b) Stroke severity GFR: Lower GFR values in relation to higher NIHSS scores. (c) LDL by severity of stroke: The LDL levels show increasing tendency with the increase in severity of strokes GFR: glomerular filtration rate; LDL: low-density lipoprotein; NIHSS: NIHSS: National Institutes of Health Stroke Scale

The overall retention of the null hypothesis across most categorical variables suggests the comparability of treatment groups and reduces concerns regarding selection bias (Figure 3). 

Logistic regression analysis

Multivariate logistic regression was used to determine predictors of recurrent stroke. The model demonstrated modest predictive power (Nagelkerke R²=0.206; p=0.232). One of the strongest predictors was smoking status: former smokers had significantly reduced odds of stroke recurrence (OR=0.331; p=0.003) compared to never smokers. History of TIA was also associated with higher recurrence risk (OR=1.884; p=0.031). Aspirin use independently reduced the odds of recurrence (OR=0.468; p=0.012). Notably, DAPT was not found to be a significant predictor in multivariable models (p=0.583), although it trended toward protective benefit. Logistic regression analysis determined that prior smoking status (OR=0.331; p=0.003), cardiovascular disease (CVD)/TIA history (OR=1.884; p=0.031), and aspirin use (OR=0.468; p=0.012) were factors that predicted stroke recurrence with clinical significance. In contrast, assignment to DAPT was not meaningfully associated with recurrence (p=0.583). These results emphasized the importance of relevant patient history and particular antiplatelet agents over treatment strategy alone when looking for outcomes.

Effect size analysis

Effect size calculations using Cohen's d supported the above findings. The difference in GFR between groups yielded a moderate effect size (d=0.38), while the LDL difference showed a small effect size (d=-0.35). Other variables, such as BMI and HDL, had minimal effect sizes (<0.2), confirming limited clinical significance.

ANOVA analysis

**Figure 4:**
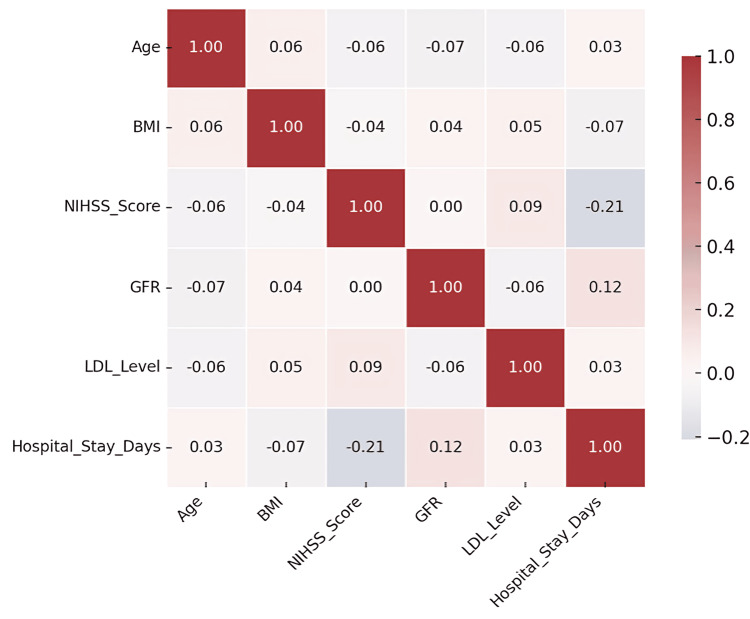
Heatmap of pairwise correlations among clinical and biochemical variables in patients with minor stroke or transient ischemic attack BMI: body mass index; NIHSS: National Institutes of Health Stroke Scale; GFR: glomerular filtration rate; LDL: low-density lipoprotein

ANOVA was used to test for mean differences in clinical parameters across stroke recurrence outcomes. Variables such as BMI, NIHSS score, blood pressure, and various laboratory measures (LDL, HDL, HbA1c, etc.) yielded non-significant p-values (all p>0.1), suggesting that these measures did not vary significantly between outcome groups (Figure 4). Although GFR (p=0.288) and HDL (p=0.132) showed minor trends, these were not statistically significant. These results suggest that univariate group-level differences in clinical or biochemical parameters are insufficient to explain outcome disparities, underscoring the need for multivariate analyses. This comprehensive inferential exploration confirms that while individual risk factors may not reach statistical significance in isolation, combined models and adjusted predictors (as in logistic regression) provide deeper insights into therapy effectiveness and outcome prediction.

## Discussion

This retrospective cohort study aimed to assess the comparative effectiveness of DAPT versus SAPT in patients with acute stroke [13]. Among the 250 patients included, 129 (51.6%) received DAPT and 121 (48.4%) received SAPT. While baseline characteristics were statistically comparable between the groups, the clinical outcomes showed nuanced trends: stroke recurrence and bleeding were slightly more frequent in the DAPT group, whereas mortality rates were nearly equivalent. However, none of these differences reached statistical significance, highlighting the clinical complexity and suggesting that neither therapy was clearly superior in this real-world cohort [14].

The primary outcome, stroke recurrence, was observed in 133 patients (53.2%). However, no statistically significant difference in recurrence was found between the DAPT and SAPT groups (p=0.312; chi-squared test), suggesting comparable effectiveness between the two strategies in this sample [15]. This finding diverges from results reported in trials such as CHANCE and POINT, possibly due to our real-world setting, smaller cohort, and retrospective design. Likewise, bleeding events occurred in 131 patients (52.4%), and mortality was observed in 127 patients (50.8%), neither of which showed significant associations with treatment arm (p=0.448 and p=0.800, respectively) [16]. Multivariate logistic regression showed that former smokers had significantly lower odds of stroke recurrence compared to never smokers (OR=0.331; p=0.003). Interestingly, former smokers were found to have significantly lower odds of stroke recurrence compared to never smokers (OR=0.331; p=0.003). This finding contradicts existing literature, which consistently indicates elevated stroke risk among both current and former smokers compared to never smokers. One possible explanation is the "healthy quitter" effect former smokers may receive more intensive follow-up or adopt healthier behaviors post-cessation. This result should be interpreted with caution and warrants further validation through prospective research. For context, Pan et al. demonstrated that both current and former smoking were associated with increased recurrence risk [17]. More prospective research studies are needed to understand this association. Conversely, a history of TIA (n=114) was a significant positive predictor of recurrence (OR=1.884; p=0.031), aligning with literature that establishes prior TIA as a marker of heightened stroke risk [18].

Interestingly, aspirin use (n=124; 49.6%) independently reduced the risk of recurrence (OR=0.468; p=0.012), while DAPT as a variable did not achieve statistical significance (p=0.583), although it showed a trend toward benefit [19]. These findings suggest that agent-specific effects, rather than the combined regimen alone, may have a more direct influence on outcomes and should guide personalized treatment plans. From a biochemical standpoint, GFR showed significant between-group differences (mean=78.99 mL/min in DAPT vs. 68.87 mL/min in SAPT; p=0.049) with a moderate effect size (Cohen's d=0.38) [20]. This implies that renal function may interact with treatment response, potentially affecting drug metabolism or complication risk. In contrast, variables like LDL (p=0.071) and HbA1c (p=0.795) did not reach significance, despite their known roles in vascular pathology [21]. The lack of statistical significance in ANOVA tests across outcome groups for most variables, that is, BMI, NIHSS, HDL, LDL, and blood pressure (all p>0.1), suggests that univariate relationships are insufficient to explain recurrence or adverse outcomes in this population. This emphasizes the importance of multivariable modeling, which identified only a few strong predictors [22].

This study has a number of strengths that support its validity and clinical relevance. The use of real-world data from a tertiary care center, and its reflections on the use of DAPT and SAPT in the context of acute stroke management/direct application in practice, supports findings from a practical standpoint. The cohort was similar across arms of treatment, and the extensive baseline and clinical data permitted a thorough statistical analysis, including multivariable logistic regression, where critical covariates, such as smoking and past TIA status, were included. The use of a demographically homogenous population allows for internal consistency, as there was limited ethnic variability. Also, ethical standards were maintained through the use of anonymized patient records and an IRB exemption (5142/AIMS/2024). These strengths lend support to the conclusions of the study and have the potential to contribute to real-world clinical decisions.

This study has several limitations that warrant consideration. As a retrospective observational analysis, it lacks randomization, introducing potential selection bias and confounding. The single-center setting in Muzaffarabad and a demographically homogenous population (n=250; DAPT=129; SAPT=121) limit the generalizability of the findings [23]. Data were extracted from medical records without standardized follow-up, increasing the risk of misclassification of outcomes like recurrent stroke or bleeding [24]. Therapy duration, adherence, and time-to-event data were unavailable, restricting the depth of outcome interpretation. Bleeding events were not stratified into major or minor, limiting assessment of safety [25]. Additionally, important biomarkers and genetic factors influencing treatment response were not evaluated. Despite these limitations, the study provides valuable real-world insights into antiplatelet therapy in acute stroke patients [26]. Furthermore, the absence of a statistically significant difference in stroke recurrence between DAPT and SAPT groups may reflect treatment selection bias. In this non-randomized study, patients at inherently higher risk, such as those with diabetes, prior stroke, or a history of smoking, were more likely to receive DAPT. This may have masked the potential benefit of dual therapy, underscoring the need for controlled prospective studies to better isolate treatment effects.

## Conclusions

This retrospective cohort study comparing DAPT and SAPT in patients with acute stroke found no statistically significant difference in recurrent stroke, bleeding, or mortality outcomes between groups (p>0.05). While DAPT showed a trend toward reduced recurrence, logistic regression revealed that aspirin use (OR=0.468; p=0.012) and former smoking status (OR=0.331; p=0.003) were stronger predictors of reduced risk. The findings underscore the complexity of stroke prevention and highlight the need for individualized antiplatelet strategies guided by clinical risk profiles and comorbidities.
